# The Relationship of Breathing and COVID-19 Anxiety When Using Smart Watches for Guided Respiration Practice: A Cross-Sectional Study

**DOI:** 10.3389/fpsyg.2022.847602

**Published:** 2022-04-25

**Authors:** Yu-Feng Wu, Mei-Yen Chen, Jian-Hong Ye, Jon-Chao Hong, Jhen-Ni Ye, Yu-Tai Wu

**Affiliations:** ^1^Office of Physical Education, Ming Chi University of Technology, New Taipei City, Taiwan; ^2^Graduate Institute of Sport, Leisure and Hospitality Management, National Taiwan Normal University, Taipei City, Taiwan; ^3^Faculty of Education, Beijing Normal University, Beijing, China; ^4^Institute for Research Excellence in Learning Sciences, National Taiwan Normal University, Taipei City, Taiwan; ^5^Graduate Institute of Technological and Vocational Education, National Taipei University of Technology, Taipei City, Taiwan; ^6^Office of Physical Education, Soochow University, Taipei City, Taiwan

**Keywords:** Apple Watch, continuance intention, COVID-19 anxiety, guided respiration practice, positive affect, positive psychology

## Abstract

COVID-19 mortality rates are increasing worldwide, which has led to many highly restrictive precautionary measures and a strong sense of anxiety about the outbreak for many people around the world. There is thus an increasing concern about COVID-19 anxiety, resulting in recommending approaches for effective self-care. From a positive psychology perspective, it is also important for people to have positive affect when dealing with this pandemic. According to previous literature, respiration is considered to be an effective way to enhance people’s mental health. Among all the wearable devices, Apple Watch has the largest market share, so this study recruited Chinese users that use respiration exercise function on Apple Watch; a total of 316 valid data were retrieved. Meanwhile, to understand one approach related to using Apple Watch to practice respiration to reduce COVID-19 anxiety about being infected during the COVID-19 outbreak, this study used a web-based cross-sectional survey to examine anxiety about being infected by COVID-19 among Chinese people who had been using the Apple Watch to practice respiration during the period of the COVID-19 outbreak. The study was based on the Health Theoretical Model, and the model was developed with four dimensions and was validated with structural equation modeling. The results of this study showed that practicing few minutes had a positive relationship on positive attitude, and positive attitude had a negative relationship on pandemic anxiety and a positive relationship on continuance use intention. Anxiety about the pandemic had a negative relationship on the intention to continue using the function. This showed that respiration practice can help to suppress the increase in anxiety levels regarding this pandemic.

## Introduction

Coronavirus infection cannot be differentiated geographically, racially, religiously, or politically and is therefore considered a global problem (Moghanibashi-Mansourieh, 2020). The COVID-19 outbreak forced many countries to quarantine, isolate, and restrict travels of their citizens to reduce potential exposure to the infection (Luis et al., 2021). In addition, the occurrence of COVID-19 and its quick spread has worsened anxiety in the global population and led to mental health disorders in individuals due to the unpredictable nature of the virus causing a continuous stressful environment (Zandifar and Badrfam, 2020). In addition, Moghanibashi-Mansourieh (2020) found that approximately one in five people experience severe and very severe anxiety. Residents of provinces with higher rates of COVID-19 infection had greater levels of anxiety, and people who score highly on threat assessments tend to overestimate the severity and likelihood of adverse events and perceive themselves as particularly vulnerable to threats (Taylor, 2019). There is no doubt that the COVID-19 epidemic has put pressure on people and society, and fear of infection has been widespread during the outbreak (Choi et al., 2020).

One study noted that the COVID-19 pandemic has impacted individuals’ mental health. To cope with the stresses and negative emotions and to seek the ability to survive and overcome this psychological harm, it is necessary to develop a positive attitude (Ardhiani et al., 2021). Many approaches have been suggested to train positive attitudes and to prevent psychological anxiety about being infected by Covid-2019 (Michels et al., 2021). For example, a few studies have focused on respiration practice to reduce COVID-19 anxiety about being infected or on positive attitude training (Ardhiani et al., 2021). Thus, the present study explored how people practice respiration using a smartwatch device.

Wang et al. (2020) investigated the psychological reactions of the Chinese community during the COVID-19 pandemic and found that 53.8% of respondents reported moderate to severe negative psychological effects from the outbreak. Therefore, there is an increasingly urgent need to understand the mental health impact of the COVID-19 pandemic in order to best prevent the occurrence of severe mental illness as a secondary consequence (Cullen et al., 2020). However, people are increasingly worried about the anxiety associated with COVID-19, suggesting the need for effective self-care and providing more mental health treatments (Peteet, 2020). Research suggests that staying mindful can help cope with stressful situations such as the one currently being experienced in this study. Respiration tends to be associated with a variety of psychological factors that can prevent emotional distress in chronic illness (Conversano et al., 2020). Moreover, some studies have shown several benefits of practicing respiration such as reducing anxiety, stress, depression (Khoury et al., 2015), and negative emotions (Sedlmeier et al., 2012), and fostering psychological well-being (Gaspar et al., 2021) and a positive attitude (Seema and Sircova, 2013). Attitude is defined as individuals’ affect, cognition, and behaviors. According to the expectancy-value theory, attitude arises according to a person’s belief about certain objects (Kato et al., 2016). Due to COVID-19, people are suffering from social alienation and isolation. Therefore, how respiration practice affects people’s positive attitude and anxiety about their health was of interest in this study.

Respiration is categorized as a method of emotion regulation, and it is believed that the processes that occur in respiration practice regulate difficult emotions that in turn contribute to physical health (Wielgus et al., 2020). In respiration practice, breathing is an exercise, relaxation, and contemplative practice, which is a respiration-based intervention that is similar to mindfulness-based stress reduction (MBSR) and mindfulness-based cognitive therapy (MBCT; Monteiro et al., 2015). Guided respiration therapy is based on the principle of integrating breathing and relaxation self-regulation (Lalande et al., 2016), which actively changes breathing behavior and sustained intensity, and thus manages the dynamic interplay of physiological and psychological processes while minimizing discomfort or cathartic expressions and increasing engagement (Lalande et al., 2017). Paying attention to the breath requires one to shift attention to the sensation of breathing and to be aware of it at all times (Chittaro and Vianello, 2016). Thus, this study aimed to explore how respiration practice *via* Apple Watch can improve positive attitude, reduce anxiety about being infected by COVID-19, and boost continuance use intention of guided respiration practice with Apple Watch.

Recently, digital software for respiration practice, including smartwatch applications (apps), has become widely available (Lau et al., 2020). The important difference between app-based respiration practice is self-directed practice, different from in-person training, which is less expensive, more time saving, and more engaging (Economides et al., 2018). On the other hand, the advantages of a respiration app include its flexibility, mobility, and ease of use, which may facilitate the probability of its use by more people (Wen et al., 2017). However, little studies have examined the effectiveness of respiration apps (Mani et al., 2015). Other studies have stated that tracking wearable devices is a trend of today’s leisure activities (Lidynia et al., 2018), which might lead to different using behavior such as how individuals adopt, use, and continue to use such devices (Canhoto and Arp, 2017). This would suggest that wearables may not be as sustainable as they may seem (Fietkiewicz and Ilhan, 2021). Therefore, it is important to determine consumers’ continuance use intention of Apple Watch for guided respiration practice. To fill this research gap, this study used a web-based cross-sectional survey to examine the perceptions of COVID-19 anxiety among Chinese people who had been using Apple Watch for guided respiration practices (for more than 6 months), their positive attitude after respiration practice, and their continuance use intention of Apple Watch for guided respiration practice during the COVID-19 pandemic.

## Methodology

### Apple Watch Assists in Practicing Respiration

According to the statistical analysis of the International Data Company IDC Corporate USA (2021), Apple’s sales of wearable devices reached 151.4 million in FY2020, accounting for 34.1% of the market, and the market share of the second largest company’s products was only 11.4%, which is sufficient to show Apple’s representativeness as a research tool. In addition, the Apple Watch offers the potential to collect and return data from individuals to provide real-time personalized feedback through the wearable’s screen (Abt et al., 2018), so this should be a suitable tool for data collection. In addition, Health and respiration are software developed by Apple and built into iPhone and Apple Watch, respectively. Respiration practice on Apple watch is a software developed by Apple in 2016 and is listed as one of the indicators of mental health by Apple. Therefore, this software was used as a research tool in this study.

**Figure 1:**
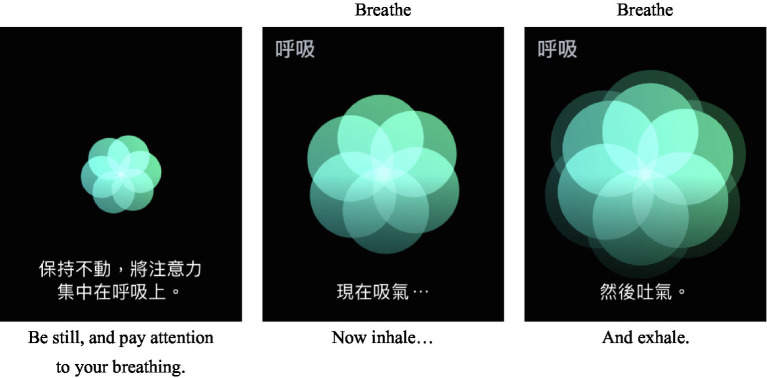
Operation method.

On the iPhone’s Health app, respiration practice time can be set as 1–5 min for the Apple Watch, and the app will ask users to focus on their breathing. In addition, the duration of breathing can be set based on inhalation and exhalation. The default value is seven times a minute, and when users breathe in, the watch face image will be enlarged and the watch will vibrate (vibration can be adjusted on users’ phone). In this way, a record will be completed. When viewing the Health app, the number of minutes of “respiration” will be recorded. In order to have a calm mind at all times, users can set the duration of respiration practice on their phone, as shown in Figure 1.

### Research Model and Hypotheses

#### Research Model

**Figure 2:**
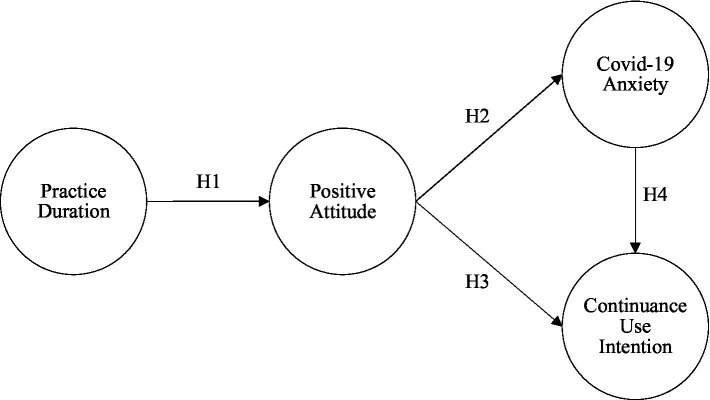
Research model.

The Health Theoretical Model (HTM; Moskowitz et al., 2019) of the positive thinking pathway suggests that when people engage in positive activities in positive psychological interventions (PPI), they increase the duration of positive emotions, thereby decreasing the production of negative emotions, and increase their engagement in preventive health behaviors. Therefore, based on this concept, this study proposed four hypothetical paths and constructed a research model, as shown in Figure 2.

#### Research Hypotheses

Self-care can be an important personal health asset to reduce the negative effects of stress caused by this condition and to help improve/maintain health (Bermejo-Martins et al., 2021), and self-care is considered an important and valuable principle because it emphasizes the active role of people in maintaining their own well-being (Martínez et al., 2021). In this study, respiration exercises were considered as a form of self-care. For example, a study has found that when performing positive interventions (especially for long periods of time) is strongly associated with an increase in positive self-attitude (Xiao et al., 2017). It is notable that some studies have proven that respiration have many positive outcomes such as reducing stress (Shearer et al., 2016; Pascoe et al., 2017) reducing perceived pain (Creswell et al., 2019), improving concentration (Norris et al., 2018), improve work memory (Mrazek et al., 2013), and improving one’s emotions (Davis and Hayes, 2011). In addition, there is evident that practice duration in respiration is highly associated with positive attitude (Bravo et al., 2018). Therefore, the following hypothesis is as follows:

*Hypothesis 1:* Practice duration has a positive relationship on positive attitude.

The COVID-19 pandemic has paralyzed people in 216 countries (regions) around the world in all aspects of life, causing panic, insecurity about life and death, fear of infection transmission, and so on is leading the field of psychology to study the power and potential of human beings as an integral part of our well-being, adaptation, adjustment, and accommodation to any adverse situation in life (Kulandaiammal, 2020). Positive psychology practices and interventions are designed to develop strengths, skills, and resources to prevent the onset of mental disorders, build resilience, and help people lead better lives (Kern et al., 2020). Among other things, guided respiration practices are considered mostly effective in terms of regulating emotions, because just observing and avoiding judgment can separate individual’s emotions, which frequently further intensify emotional responses (Guendelman et al., 2017).

There is evidence that practicing respiration reduces harmful health behaviors (Salmoirago-Blotcher et al., 2013) and can improve mental behavior (Gawande et al., 2019; Schuman-Olivier et al., 2020). Studies have also found that having a positive attitude helps people fight anxiety and will lead them out of their darkest times (Chundawat, 2018). Another study related to attitude, anxiety, and practices related to COVID-19 among Jordanian University students found that students had positive attitudes toward COVID-19 anxiety (Olaimat et al., 2020); what this implies is that individuals’ positive attitude may prompt them to adopt preventative behaviors (Roy et al., 2020; Torales et al., 2020). Given that respiration practices can improve individuals’ positive attitudes toward COVID-19 anxiety, it could be the key to controlling anxiety. Therefore, the following hypothesis was proposed:

*Hypothesis 2:* Positive attitude has a negative relationship on COVID-19 anxiety.

Studies have noted that attitudes can be identified as positive or negative beliefs about a product, as a result of having direct experiences of the product (Amoroso and Lim, 2017). Oliver (1980) proposed a cognitive theory in which he argued that an individual’s behavioral intentions can be conceptualized through attitudes. According to the Technology Continuous Theory (TCT), attitude is the main driver of users’ continuous desire (Daragmeh et al., 2021). Previous studies have also indicated that users are found to have a positive attitude when they believe that the technology they use will improve their productivity (Chau and Hu, 2001). Attitude has been found to be a strong determinant of continuance intention (Black, 2005) and other studies related to technology have also confirmed that attitude has a strong impact on continuance use intention (Amoroso and Ogawa, 2011; Cheng, 2011). Therefore, the following hypothesis was proposed:

*Hypothesis 3:* Positive attitude has a positive relationship on the desire for continuance use intention.

Anxiety is found especially during pandemic, and this may influence individuals’ behavior (Roy et al., 2020). This behavior includes users receiving tailored analyses of their health status and physical activity, so health-seeking users are increasingly self-measuring. We therefore hypothesized that high self-quantifiers will become more attached to the technological devices on their wrists (Siepmann and Kowalczuk, in press). Individuals with higher anxiety about COVID-19 will have a negative affective reaction toward technology use due to their anxious feelings, and their anxiety will weaken their continuance use intention (Venkatesh, 2000). Having high anxiety leads to a decrease in sensory and motor systems (Xue et al., 2012), which makes it harder for individuals to use Apple Watch for guided respiration practice. In other words, when people do not feel less anxious after using respiration app, they may be less inclined to continue using it. Therefore, the following hypothesis was proposed:

*Hypothesis 4:* COVID-19 anxiety has a negative relationship on continuance use intention.

### Data Collection

Participants were recruited using a snowball sampling method, and the questionnaire link and QR code were distributed to community members on social media, Weibo, and WeChat (the most popular messaging app in China) who shared their daily positive thoughts and growth, so that all Chinese using WeChat or other social networking tools could see the survey. On the first page of the online questionnaire, this study first explained the purpose, the object for filling in the data, the purpose of the data, and anonymous information. Although this is a self-reported experience, we believe that those participants willing to assist had performed guided respiration practice *via* smartwatches for more than 6 months. When participants completed the survey anonymously, they were encouraged to forward the link to others they knew. Data were collected from 1 to 30 November 2020. In the data collection process, the focus of this study was on those who had at least 6 months of experience in respiration exercises. Therefore, people who subjectively perceive whether they are anxious or not were confirmed through *post-hoc* statistical analysis that Covid-19 anxiety patients were not included in this study.

### Participants

A total of 374 questionnaires were collected in this study. In the 374 collected data, when the participant completing in the questionnaire within 3 min or when the answers to the questions were outliers in the same dimension, this study questioned the validity of these data, so the 58 data that belonged to these criteria were deleted, resulting in a valid sample size of 316, with a valid recall rate of 84.5%. There were 132 male participants (41.8%) and 184 female participants (58.2%). There were 140 respondents (44.3%) aged 20 or older but under 30, 58 (18.4%) aged 30 or older but under 40, 77 (24.4%) aged 40 or older but under 50, and 41 (12.9%) aged 50 or older.

### Instruments

Confirmatory factor analysis (CFA) is the best choice for investigating the structural validity of the scale when there are strong theoretical assumptions about the scale structure, and CFA can be used to identify the general structure of the instruments (Raju et al., 2002). This study was a quantitative validation study in which data were collected through the questionnaires (positive attitude, COVID-19 anxiety, and continuance use intention) and was developed from the concepts of previous studies and reviewed by 3 positive psychology experts to confirm the face validity of the questionnaires. The expert review was conducted in three phases: the first phase of the review focused on the appropriateness and completeness of the design of the vector and its topics, and suggested corrections; the second phase of the review focused on the readability of the revised topics and suggested corrections; and the third phase of the review focused on the fluency of the revised topics and suggested corrections. Finally, five users who had used the Apple Watch’s respiration practice were asked to take a trial reading of the scale. The questionnaires content was measured on a five-point Likert scale (1–5 for strongly disagree to strongly agree).

#### Positive Attitude

A positive attitude is a guide to living a positive life, so preserving a positive attitude from the ups and downs will be vital in all aspects of life (Chundawat, 2018). Based on the above concepts, this study developed a construct of positive attitude with nine questions to measure the degree of positivity of participants’ attitude in response to different negative events. For example, “When I face a setback, I have to overcome it in a different way” and “When things get tough in my life, I think it’s time for me to grow more.”

#### COVID-19 Anxiety

The main purpose of this study was to examine participants’ perceptions of anxiety about COVID-19, and participants assessed their experience of this specific anxiety on the basis of COVID-19 (Lee, 2020). Based on the above concepts, this study developed the construct of COVID-19 anxiety, with eight items, where each item was completed to investigate the unique manifestation of this particular form of anxiety, in order to measure participants’ perceptions of anxiety about being infected during the COVID-19 epidemic. For example, “I am concerned that I may have been infected as a result of the spread of the epidemic” and “I would be worried that I might be infected sooner or later, as in the case of the current outbreak.”

#### Continuance Use Intention

By wearing a smartwatch continuously, users can benefit from the available features as it encourages them to lead healthier lifestyles (Siepmann and Kowalczuk, in press). With this concept in mind, the present study adapted the Product Continuance Intention Scale designed by Hong et al. (2017) with six items to measure the participants’ intention to continue using the smartwatch for guided respiration practice. For example, “In the future, I would like to use a smartwatch to assist in the practice of respiration” and “I would like to continue to use my smartwatch in the future when it has new content for respiration exercises.”

#### Practice Duration in Minutes

The number of practice duration used in this study is the average number of minutes over 6 months’ participants used guided respiration practice *via* Apple Watch. These data were obtained from the average number of minutes of “respiration practice” recorded in the participant’s “health app,” as provided by the participant. The results will be calculated in mean (number of minutes) of weekly practice for the participants and minimum value in minutes and a maximum value in minutes of practice will be included. In addition, this study will also indicate the mean value (minutes) and SD (minutes) of participants that practice respiration.

### Data Analysis and Procedures

This study adopted Structural equation modeling (SEM) for the study model validation to investigate the relationship between positive attitude, COVID-19 anxiety, continuance use intention, and practice duration in minutes. In general, each SEM analysis will go through the steps of model specification, data collection, identification, parameter estimation, data-model fit assessment, model estimation, and model evaluation, but there may be a need for model modification (Mueller, 1997; Lei and Wu, 2007). Based on this statistical convention, this study collected data by modeling and formulating research hypotheses through literature and theory, and then coded and organized the data. Item analysis was used to confirm the fit of each dimension in sequence, and then reliability and validity analysis is carried out. After confirming that the dimension has good reliability and validity, the fit of the research model is carried out. In addition, when the model had a good degree of fit, model validation was then conducted, and the complete validation analysis results are as follows.

### Item Analysis

The item analysis in this study was conducted using first-order confirmatory factor analysis. Hair et al. (2019) suggested that the *χ*^2^/*df* value should be less than 5, the RMSEA should be less than 0.10, and the GFI and AGFI should be higher than 0.80. Those items with a factor loading lower than 0.50 should be deleted from the original questionnaire; after the first-stage CFA, the deletion result of positive attitude was that the number of items was reduced from nine to seven; COVID-19 anxiety was reduced from eight to seven; and continuance use intention was reduced from six to five (Table 1). The factor loadings of the deleted items ranged from 0.59 to 0.79, and the *t*-values ranged from 13.61 to 19.81, which met the criteria proposed by Green and Salkind (2004).

**Table 1:** First-order confirmatory factor analysis (CFA).

Index	*χ* ^2^	*df*	*χ*^2^/*df*	RMSEA	GFI	AGFI	FL	*t*
Value	–	–	<5	<0.10	>0.80	>0.80	>0.50	>3
Positive attitude	33.5	14	2.39	0.07	0.94	0.94	0.59–0.70	13.61–16.52
COVID-19 anxiety	28.8	14	2.06	0.06	0.99	0.98	0.68–0.79	16.80–19.81
Continuance use intention	5.7	5	1.14	0.02	0.99	0.98	0.59–0.71	16.40–18.45

### Reliability and Validity Analysis

The internal consistency Cronbach’s *α* value of 0.78–0.90 and the composite reliability value of 0.78–0.90 exceeded the value of 0.70 suggested by Hair et al. (2019), indicating that the constructs had good reliability. The factor loadings ranged from 0.65 to 0.85 and Average Variance Extracted from 0.42 to 0.56, which met the criteria proposed by Fornell and Larcker (1981). When the CR value is greater than 0.6 and AVE is higher than 0.4, it indicates that the construct has validity (Table 2).

**Table 2:** Reliability and validity analysis.

Construct	*M*	*SD*	*α*	*FL*	CR	AVE
Positive attitude	3.71	0.61	0.84	0.65	0.84	0.42
COVID-19 anxiety	3.19	0.81	0.90	0.74	0.90	0.56
Continuance use intention	3.45	0.71	0.78	0.64	0.78	0.43

## Results and Discussion

The data of this study were analyzed by item analysis, reliability and validity analysis, to confirm the construct validity. The model fit analysis was analyzed to confirm the suitability of the model, and then carry out model verification.

### Model Fit Analysis

In order to confirm the suitability of the model, statistical experts recommend that the *χ*^2^/*df* value should be less than 5 in the model fitness analysis (Hair et al., 2019), the RMSEA value should be less than 0.10, GFI, AGFI, NFI, NNFI, CFI, IFI, and RFI values should be greater than 0.80 (Abedi et al., 2015), and PNFI and PGFI equivalents should be greater than 0.50 (Hair et al., 2019). The fitted index values for this study were *χ*^2^ = 250.9, *df* = 167, *χ*^2^/*df* = 1.50, RMSEA = 0.04, GFI = 0.93, AGFI =0.91, NFI = 0.90, NNFI = 0.96, CFI = 0.97, IFI = 0.97, RFI = 0.89, PNFI = 0.79, and PGFI = 0.74.

### Path Analysis

**Figure 3:**
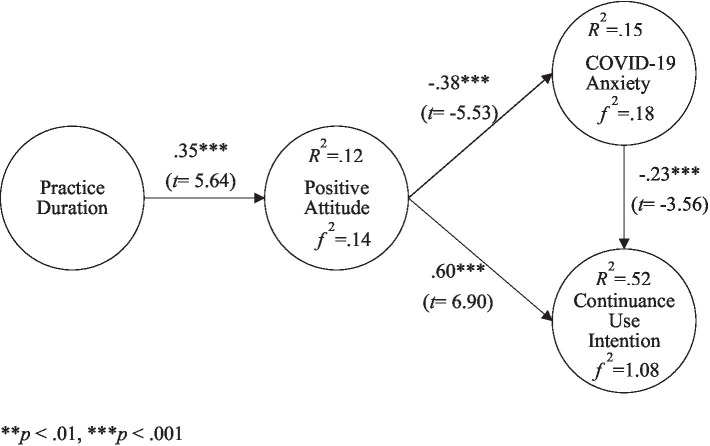
Validation of the research model.

Model validation results show that had practice duration had a positive relationship on positive attitude (*β* = 0.35***; *t* = 5.64); positive attitude had a negative relationship on COVID-19 anxiety (*β* = −0.38***; *t* = −5.53); positive attitude had a positive relationship on continuance use intention (*β* = 0.60***; *t* = 6.90); and COVID-19 anxiety had a negative relationship on continuance use intention (*β* = −0.23***; *t* = −3.56), as shown in Figure 2. The explanatory power of practice duration for positive attitude was 12% and f2 was 0.14; The explanatory power of positive attitude for COVID-19 anxiety was 15% and f2 was 0.18; The explanatory power of positive attitude and COVID-19 anxiety on continuance use intention was 0.52% and f2 was 1.08, as shown in Figure 3.

### Analysis of Practice Duration in Minutes

In this study, descriptive statistical analysis of guided respiration practice was conducted using SPSS 23.0 for Windows. The results of the analysis showed that the mean number of minutes of weekly practices for the participants in this study had a minimum value of 10 min and a maximum value of 60 min, with a mean value of 34.92 min and a SD of 12.50 min, as shown in Table 3.

**Table 3:** Analysis of the average number of weekly practices in minutes.

Construct	Minimum value	Maximum value	Mean	SD
Practice duration	10	60	34.92	12.50

### Discussion

Any new pandemic has the potential to cause considerable anxiety, and although, similar to other stressor emotions, pandemic-related anxiety may disappear without any intervention, more intensive interventions should be offered to people suffering from severe pandemic disease-related anxiety (Choi et al., 2020). However, at this critical time, it is essential that anxiety be managed in a positive manner (Goodwin et al., 2020) to address pandemic-related mental health problems. Therefore, maintaining people’s mental health is an important part of the fight against COVID-19 anxiety (Dubey et al., 2020). Several studies have shown that when performing positive intervention such as respiration for a period of time is positively related with individuals’ positive attitude practicing respiration can help improve individuals’ positive attitude (Xiao et al., 2017). These positive attitude includes improvement or maintaining health (Bermejo-Martins et al., 2021), maintaining well-being (Martínez et al., 2021), reducing stress (Shearer et al., 2016; Pascoe et al., 2017), reducing perceived pain (Creswell et al., 2019), improving concentration (Norris et al., 2018), and improving one’s emotions (Davis and Hayes, 2011). This study is consistent with previous studies and has shown that practice duration may improve individuals’ positive attitude (Bravo et al., 2018).

This study found that respiration practices can improve individuals’ positive attitudes toward COVID-19 and that it is key to controlling anxiety. This study echoes a previous study which found that individuals with higher positive attitude had lower anxiety (Chundawat, 2018). Therefore, positive attitude plays an important role in COVID-19 anxiety as stated in previous studies, where authors found that health professionals with higher positive attitudes towards pandemics will show lower levels of anxiety (Olaimat et al., 2020). Moreover, this study found that participants who used Apple Watch for guided respiration practice had positive attitudes, which implies that they found that respiration practice *via* Apple Watch improved their attitude (Chau and Hu, 2001).

Previous study has showed that individual can perceive positive or negative attitude toward a product after experiencing the product (Amoroso and Lim, 2017). This implies that individuals’ behavioral intention can be conceptualized through attitude (Oliver, 1980). Therefore, attitude is the one of the main drivers of users’ continuous desire (Daragmeh et al., 2021). In addition, this study echoes with previous studies which found that attitude had a strong impact on continuance intention (Black, 2005; Amoroso and Ogawa, 2011; Cheng, 2011). Thus, the participants of this study have positive attitude in using Apple Watch for guided respiration and are willing to continue using the product.

A study by Wang et al. (2020) indicated that 53.8% of the Chinese people surveyed had moderate to severe negative psychological effects. In the present study, the respondents showed moderate levels of COVID-19 anxiety (*M* = 3.19, *SD* = 0.81). In addition, previous studies have suggested that respiration-based psychological training can be an effective intervention (Conversano et al., 2020), and previous studies have also demonstrated that respiration can reduce anxiety, and suggested the need to develop and implement appropriate respiration-based therapeutic interventions (Dubey et al., 2020). In addition, changes in mental health outcomes were largely dependent on the duration of app use, with those who used the respiration app more frequently during the 30 days of free access demonstrating the greatest effectiveness in terms of respiration and, to a lesser extent, anxiety states (Flett et al., 2019). This study also found that the more frequently people used Apple Watch for respiration practice, the lower their perceived levels of COVID-19 anxiety, suggesting that the results of this study echo those of previous research.

It is interesting to point out that COVID-19 anxiety has a negative relationship on continuance use intention. This could be because, for an individual to practice respiration, relaxation, and contemplative practice is required (Monteiro et al., 2015). However, participants with higher anxiety about COVID-19 might have a negative affective reaction toward technology use; this is due to anxious feelings and anxiety that weakens their continuance use intention (Venkatesh, 2000). This result can also be deduced from the fact that people do not feel less anxious after using the respiration app, which makes them less willing to continue using it.

## Conclusion and Recommendations

### Conclusion

This study is a cross-sectional study in which causality may not be confirmed, but this study uses a validated factor analysis to elucidate the correlation between potential user influences and the measured variables. Therefore, at the peak of the epidemic, a questionnaire was administered to users who had been using the Apple Watch for 6 months to perform guided respiration practice to understand the subjective perceptions of these users.

Based on the above this study also confirmed that, within the framework of HTM, practice duration had a positive relationship on positive attitude. Positive attitude had a negative relationship on COVID-19 anxiety and a positive relationship on continuance use intention. COVID-19 anxiety had a negative relationship on continuance use intention regarding the feature. The contribution of this study is that the use of Apple Watch for guided respiration may help with stress during COVID-19 pandemic. The guided respiration practice was shown to have positive benefits on psychological states during the COVID-19 pandemic.

In addition, Wearable health technologies (WHT) in the form of smartwatches show promise for improving people’s mental health as they promote health based on autonomy while also providing quantitative data that allow people to quickly read their exercise information and other physiological information [e.g., heart rhythm, respiratory rate, and electrocardiography (ECG)]. Therefore, after using the smartwatch for guided respiration practice, users can also get more health information from the recorded information.

### Implications

Apps allow people to practice respiration anywhere, and they can also help beginners learn different types of respiration without spending a great deal of time or effort. Past research has found that respiration apps are effective in terms of reducing symptoms of anxiety. Also, as with many new therapies, there is growing interest in using wearable devices for mental health. Therefore, this study indicated that during the COVID-19 pandemic, the Apple Watch could be used to facilitate guided respiration practice that is convenient and available anywhere. It is also the first study to demonstrate the benefits of using a wearable smart watch to guide respiration with respiratory practice for positive psychology.

In addition, although the use of self-reporting tools and social expectations may influence the results, the validity of using online data collection was also confirmed in this study by conducting hypothesis testing through structural equation modeling under the influence of pandemic where it is difficult to invite participants to conduct physical experiments. Therefore, the use of self-reported data versus online data collection during a pandemic remains a compromise solution.

### Limitations and Future Study

In this study, three new questionnaires were developed and compiled, and although the statistical results have good reliability and validity, this study is a validated factor analysis study and it is not possible to know how inferable these three subquestionnaires are. Therefore, in the follow-up study, it is suggested that the same mature instrument can be used as a calibration validity study, and the reliability and validity of the three questionnaires can be compared with each other for participants from different countries.

As this study features a self-reported survey study conducted during the outbreak which was not conducted in an experimental setting, future studies may collect data from real respiration practice in a lab experiment to explore how the lab practice environment effects on respiration. Future research can compare this study with experimental studies to see if the two different types of study results are the same.

When recruiting participants for this study, emphasis was placed on those with at least 6 months of experience in breathing exercises, and there was no restriction on whether people were subjectively perceived as anxious or not. Therefore, in future studies, if the results of this study are to be revalidated, it is suggested that the study design can be divided into two phases. In the first phase, participants with high levels of anxiety were recruited through an instrumental assessment, and in the second phase, the experimental manipulation of time series analysis should be used to further understand the causal relationship between respiration exercises in Apple Watch and these constructs.

In addition, because we adopted an online participant recruitment method, it was not possible to actually meet the participants, making it easy to have participant attrition. This study therefore used a one-time cross-sectional survey method, but future studies can conduct longitudinal research to determine changes in participants’ levels of anxiety perception.

## Data Availability Statement

The raw data supporting the conclusions of this article will be made available by the authors, without undue reservation.

## Ethics Statement

Ethical review and approval was not required for the study on human participants in accordance with the local legislation and institutional requirements. Written informed consent for participation was not required for this study in accordance with the national legislation and the institutional requirements.

## Author Contributions

Y-FW, M-YC, J-HY, J-CH and J-NY: concept, design and drafting of the manuscript. J-HY, J-CH and J-NY: acquisition of data and statistical analysis. Y-FW, M-YC and Y-TW: critical revision of the manuscript. All authors contributed to the article and approved the submitted version.

## Funding

Ministry of Education (MOE) and Ministry of Science and Technology in Taiwan.

## Conflict of Interest

The authors declare that the research was conducted in the absence of any commercial or financial relationships that could be construed as a potential conflict of interest.

## Publisher’s Note

All claims expressed in this article are solely those of the authors and do not necessarily represent those of their affiliated organizations, or those of the publisher, the editors and the reviewers. Any product that may be evaluated in this article, or claim that may be made by its manufacturer, is not guaranteed or endorsed by the publisher.
